# A method to achieve homogeneous dispersion of large transmembrane complexes within the holes of carbon films for electron cryomicroscopy

**DOI:** 10.1016/j.jsb.2013.01.004

**Published:** 2013-04

**Authors:** Martin Cheung, Naoko Kajimura, Fumiaki Makino, Masamichi Ashihara, Tomoko Miyata, Takayuki Kato, Keiichi Namba, Ariel J. Blocker

**Affiliations:** a Schools of Cellular and Molecular Medicine and Biochemistry, Medical Sciences Building, University of Bristol, University Walk BS8 1TD, United Kingdom; b Graduate School of Frontier Biosciences, Osaka University, 1-3 Yamadaoka, Suita, Osaka 565-0871, Japan; c Riken Quantitative Biology Center, 1-3 Yamadaoka, Suita, Osaka 565-0871, Japan

**Keywords:** Electron cryomicroscopy, Single particle, Transmembrane protein complexes, Detergents, Methodology

## Abstract

Difficulties associated with using X-ray crystallography for structural studies of large macromolecular complexes have made single particle cryo-electron microscopy (cryoEM) a key technique in structural biology. The efficient application of the single particle cryoEM approach requires the sample to be vitrified within the holes of carbon films, with particles well dispersed throughout the ice and adopting multiple orientations. To achieve this, the carbon support film is first hydrophilised by glow discharge, which allows the sample to spread over the film. Unfortunately, for transmembrane complexes especially, this procedure can result in severe sample adsorption to the carbon support film, reducing the number of particles dispersed in the ice. This problem is rate-limiting in the single particle cryoEM approach and has hindered its widespread application to hydrophobic complexes. We describe a novel grid preparation technique that allows for good particle dispersion in the ice and minimal hydrophobic particle adhesion to the support film. This is achieved by hydrophilisation of the carbon support film by the use of selected detergents that interact with the support so as to achieve a hydrophilic and neutral or selectively charged surface.

## 0 Introduction

The popularity of cryoEM for structural analysis of macromolecular assemblies is due to the speed and ease of the data collection procedure. Difficulties associated with crystallisation have limited application of X-ray crystallographic techniques to a handful of large complexes. For membrane-derived macromolecular complexes, which show an even lower propensity to crystallise, cryoEM is often the only option (Chandran et al., 2009; Lau and Rubinstein, 2012; Yusupov et al., 2001).

Single particle cryoEM involves the suspension of macromolecules in vitrified ice, acquisition of data at low temperature and low electron dose and computerised processing of the images. This technique retains the complexes in a hydrated state, giving a good representation of their native structure. Due to technical advances, the achievable resolution is now sub-nanometre (Jiang and Ludtke, 2005).

For cryoEM, the highest quality images, in terms of contrast, signal to noise ratio (S/N) and resolution, are attained when the sample is applied to a holey carbon support film and particles are dispersed in the ice within the holes of the carbon. However, continuous carbon support films are often used because particles do not distribute efficiently into the holes. This improves particle spread but limits the observable particle orientations. The carbon also makes the image contrast of the particles lower, impairing high-accuracy alignment required for extraction of high-resolution image information.

## 1 Consequences of grid preparation by glow discharge

Carbon support films are generally hydrophilic immediately after production. However, adsorption of organic molecules and/or oil vapour from the environment gradually diminishes surface charges, eventually rendering support films hydrophobic (Dubochet et al., 1985; Sogo et al., 1975). To achieve spreading of the sample over the grid, the grid surface is first hydrophilised by ion bombardment. In a reduced air environment, glow discharging creates a hydrophilic surface by removal of adsorbed impurities and deposition of electrons onto the carbon surface, thereby creating a negatively charged surface (Dubochet et al., 1985; Hayat, 2000). Charging the grid surface also increases charge interactions between the carbon and protein, further aiding sample spreading.

The key difference between holey and continuous carbon supports is the role of the carbon. With continuous supports, the carbon acts as a support for the sample itself. Therefore, anything that increases the interaction between the support and sample is advantageous. However, for holey carbon supports, the carbon and holes provide an aqueous environment for the sample. The goal is to have particles dispersed within thin sample solution films formed in the holes before freezing. Here, anything that increases interactions between the support and sample results in sample adsorption to the carbon support, inhibiting particle dispersion within the holes.

## 2 Transmembrane macromolecular complexes and cryoEM

In an aqueous environment, macromolecules exist in an ionised state, with the degree of ionisation dependent on buffer constituents, solution pH, temperature etc. For this reason, macromolecular complexes often adsorb strongly onto hydrophilic surfaces (Dubochet et al., 1985). Transmembrane protein complexes are removed from lipid bilayers by solubilisation with a detergent, resulting in large protein–detergent complexes. Along with vast hydrophobic regions, all transmembrane complexes will have hydrophilic regions exposed to the cytosol, periplasm and/or extracellular space. The complexity of surface charges of the solubilised complexes further exasperates adsorption onto hydrophilic surfaces. Sample adsorption to glow discharged holey carbon films prevents particle dispersion within the holes and has become a common obstacle in cryoEM studies of transmembrane macromolecular complexes.

## 3 Principle of the new method

The propensity of transmembrane complexes to adsorb to glow discharged carbon surface may indicate a problem not with glow discharge *per se*, but with surface ionisation in general. Any method that serves to increase surface hydrophilicity by inducing ionisation is likely to have the same adsorption effect on transmembrane complexes. With samples where the adsorption to the support film is almost complete, it is often better to apply the sample to a non-glow discharged, hydrophobic grid as this may yield at least some dispersed particles. However, a more logical workaround would be to create a hydrophilic surface with an overall neutral or selected charge. To achieve such a surface, we thought to pretreat the carbon support with detergents.

The amphipathic nature of detergent molecules makes them ideal for altering the properties of a surface. If applied to a hydrophobic surface, a detergent molecule should orient in the most energetically favourable manner, i.e. with the non-polar tail group facing the surface and the polar head group facing away from the surface. The wide variety of commercially available detergents with differing head group polarities (non-ionic, anionic, cationic and zwitterionic) allows one to obtain a surface charge that best suits the requirements of the specimen.

## 4 Grid types used

We purchased carbon-coated grids, with a defined hole size (R 0.6/1, Molybdenum, 200 mesh; Quantifoil Micro Tools GmbH, Germany). Newly purchased grids are assumed to be hydrophobic, and any ionisation of the carbon support should be avoided to assure this. For reproducibility, impurities from the manufacturing process were removed by incubating grids overnight on filter paper soaked in chloroform, with the carbon side facing away from the filter paper. This was followed by an overnight wash in toluene, using the same procedure. This wash was conducted in advance and the grids stored under reduced-humidity conditions.

## 5 Detergent selection and concentration

Detergent molecules in solution form micelles once the detergent concentration surpasses the critical micelle-forming concentration (CMC). At any detergent concentration above the CMC, the concentration of detergent monomers is roughly equal to the CMC, meaning the number of free monomers is essentially constant regardless of concentration (Kaufmann et al., 2006; Prive, 2007). Only detergent monomers are likely to become adsorbed to a hydrophobic surface due to the availability of the tail group. A micelle is unlikely to bind to a hydrophobic surface since tail groups are sequestered within its core. Therefore, the precise detergent concentration is unlikely to be important as long as the CMC is exceeded. Indeed, for *n*-dodecyl-*β*-D-maltopyranoside, we tested concentrations 10 and 100-fold above the CMC and found that they gave similar results. However, to minimise the risk of detergent contamination of the sample, the concentration should probably best not vastly exceed the CMC.

We tested a non-ionic detergent, *n*-dodecyl-*β*-D-maltopyranoside (DDM, CMC ≈ 0.17 mM, 0.009% (w/v)); a zwitterionic detergent, *N*,*N*-dimethyldodecylamine-*N*-oxide (LDAO, CMC ≈ 1 mM, 0.023% (w/v)) and a cationic detergent, Cetryltrimethylammonium Bromide (CTAB, CMC ≈ 1 mM, 0.036% (w/v)). Their structures and properties are summarised in Table 1. A final detergent concentration of 0.1% (w/v) was used for each detergent, which represents detergent concentrations of approximately 2 mM, 4 mM and 3 mM, respectively.

## 6 Grid pretreatment procedure

Prior to conducting sample vitrification, a 10% (w/v) stock solution of the chosen detergent is made in distilled water. Untreated grids are then floated, carbon support face down, on the surface of a 0.1% detergent solution. The grid(s) are floated on the detergent solution at 25 °C for 1 to 12 h. Two hours before sample vitrification, three 10 μl drops of distilled water per grid are placed on a sheet of parafilm. Using tweezers to grasp the edge, a grid is removed from the detergent solution. Filter paper is used to blot away excess detergent solution by touching the grid edge, without touching the carbon surface itself. Blotting should continue until no more detergent solution is absorbed by the filter paper, but without letting the grid become dry (<15 s). The grid is placed with its carbon face down into the first drop of water, left for 2 s and then removed. The excess liquid on the grid is blotted away as before. This procedure is repeated for the remaining two drops of water. After washing, the grid(s) are placed on a sheet of filter paper placed inside a covered Petri dish, with carbon side facing up. The grid(s) are left to dry at room temperature until vitrification (∼1.5–2 h). As with ionised grids, contaminants in the atmosphere will adsorb onto the surface of detergent-coated grids, gradually diminishing the effect of the treatment. It is therefore advisable to complete the procedure 1.5–2 h before vitrification to allow sufficient time for the grid(s) to dry whilst limiting their exposure to air.

## 7 Samples tested

Initial experiments were performed with:

(i) The needle complex (NC) from *Shigella flexneri* (Zenk et al., 2007). A complex spanning the bacterial cytoplasm and periplasm, embedded in the inner and outer membranes, from which a 50 nm needle extends into the extracellular space. It is used to transfer proteins directly from the bacterium into the host cell, resulting in cellular invasion;
(ii) the FliF ring of the bacterial flagellum (Suzuki et al., 2004). The FliF ring is the inner membrane ring of the flagellar HBB and the core structure of the rotary motor as well as the base that initiates the entire flagellar assembly;
(iii) the flagellar hook-basal body (HBB) with short filaments attached, from *Salmonella enterica* serovar Typhimurium (Makino et al. in preparation). The HBB is a large transmembrane complex, spanning both inner and outer membranes, with the hook and filament being extracellular portions. This is responsible for bacterial motility.

However, all figures shown and quantifications detailed below are from HBBs with short filaments attached.

## 8 Microscopy conditions

Sample vitrification was performed using a semi-automated vitrification device (Vitrobot, FEI). A 3 μl of sample solution was applied to the detergent pretreated EM grid in the Vitrobot at 90–100% humidity, 4 °C. The grid was then automatically blotted once from both sides with filter paper by using a 3 s blot time. The grid was then plunged into a liquid ethane with a 0 s delay time. Specimens were observed with either of two types of JEM3200FSC electron microscopes (JEOL). Both are equipped with a liquid-nitrogen-cooled specimen stage, an Ω-type energy filter, and a field-emission electron gun operated at 200 kV. Zero energy-loss images, with a slit setting to remove electrons of an energy-loss larger than 10 eV, were recorded with an electron dose of approximately 20 electrons/Å^2^, either on a 4096 × 4096 15 μm/pixel slow-scan CCD camera, TemCam-F415MP (TVIPS), at a magnification of around 20,000×, and a defocus range of 4.0–5.0 μm (G4 microscope) or on a 8192 × 8192 15.6 μm/pixel CMOS camera, TemCam-F816 (TVIPS), at a magnification of around 50,000×, and a defocus range of 3.0–4.0 μm (G6 microscope).

## 9 Glow discharged grids cause dramatic adsorption of membrane protein complexes to the carbon support

**Fig.1:**
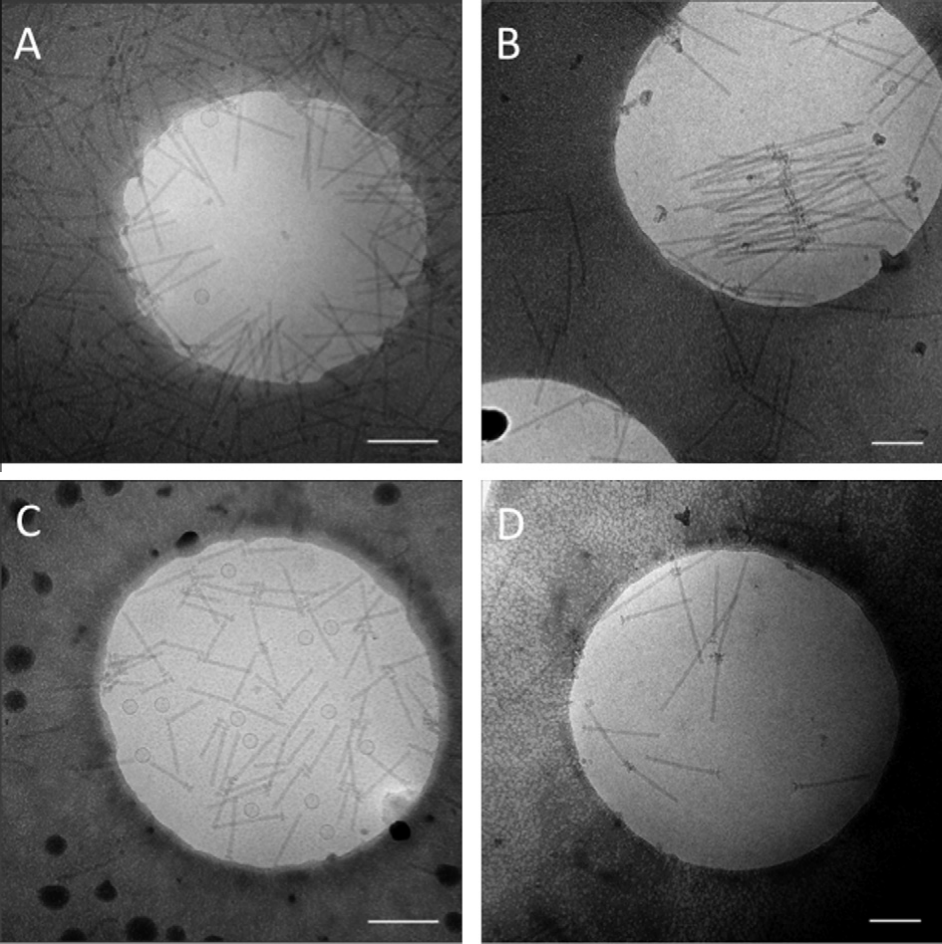
HBB samples applied to glow discharged grid and grids pretreated with various detergents. (A) Glow discharged grid – The majority of HBBs adsorbed to the carbon support film. (B) DDM pretreated grid – The adsorption of HBBs onto carbon surface was significantly reduced compared with glow discharged grids. However, DDM pretreatment moderately aggravated the aggregation of HBBs. (C) CTAB pretreated grid – A high concentration of well dispersed HBBs were seen, with next to no attachment to carbon. (D) LDAO pretreated grid – Grids treated with LDAO showed a remarkable reduction in particle adsorption to the carbon, but fewer particles were also found the holes themselves. Scale bars represent 200 nm.

All three complexes tested showed significant adsorption to the carbon support when grids were glow discharged prior to sample application. NCs showed dramatic adsorption to the carbon, with few dispersed particles seen. A large number of NCs were bound to the hole edges. Large aggregates of degraded protein were also commonly found attached to the hole edges. The FliF ring sample also adsorbed to glow discharged grids strongly. As was commonly the case for NCs, a population gradient of predominantly aggregated particles was seen from the carbon support to the centre of the grid holes, where almost no particles were found. HBBs interacted with the carbon support almost exclusively through their hydrophobic basal body portion. Almost no dispersed HBBs were seen, with the majority of particles bound to the surface of the support film (Fig. 1A).

## 10 DDM pretreatment decreases membrane protein binding to the carbon surface

Grids pretreated with DDM showed a moderate increase in dispersion of NCs compared to glow discharged grids. The vast majority of the protein complex still adsorbed onto the carbon. A stronger but similar effect of DDM-treated grids was seen with HBBs. However, in addition, HBBs also showed an increase in aggregation through their hydrophobic base portion (Fig. 1B).

## 11 CTAB has variable effects on membrane protein complex adsorption to the carbon surface

Pretreating non glow-charged grids with the cationic detergent CTAB did not improve NC dispersion. In fact, the CTAB coat seemed to increase the amount of aggregated NCs bound to the hole edges. There was also little reduction in the amount of NCs adsorbed to the surface of the carbon support. NCs applied to a non-glow discharged (hydrophobic) grid results in a low number of particles within the holes or adsorbed to the carbon. Therefore, the increased amount of aggregated NCs seen with CTAB-treated grids indicates a genuine effect of the detergent. However, CTAB treated grids allowed the vast majority of applied HBBs to disperse within the holes rather than adsorb to the carbon surface (Fig. 1C).

## 12 LDAO pretreatment largely prevents membrane proteins from binding to the carbon surface

With LDAO pretreated grids, excellent NC dispersion was seen. Although some particles were still attached to the hole edges, few NCs adsorbed to the carbon surface itself. Where NCs adsorbed onto the carbon surface, it appeared to be mainly aggregates. The number of dispersed FliF rings was also vastly improved. A significant reduction in protein adsorption to the carbon surface was also seen. Dispersion of HBBs was also improved with LDAO-treated grids, with the majority of HBBs appearing dispersed through the ice. Only occasionally were HBBs seen attached to hole edges or adsorbed onto the carbon surface (Fig. 1D).

All detergents increase the percentage of particles in the holes but some lead to higher numbers of particles per hole than others.

A quantification of the number of HBB particles within grids holes (versus on the carbon) under the different conditions tested showed that, for glow-discharged grids, only ∼20% of the particles were found within the holes while 70–90% of particles were found within the holes for all detergent-treated grids tried. However, we also noted that, for HBBs, LDAO- and to a lesser extent DDM-pretreatment led to fewer particles seen per hole than CTAB-pretreatment (Fig. 1).

## 13 Detergent treatment of grids probably increases sample spreading by surface wetting

**Fig.2:**
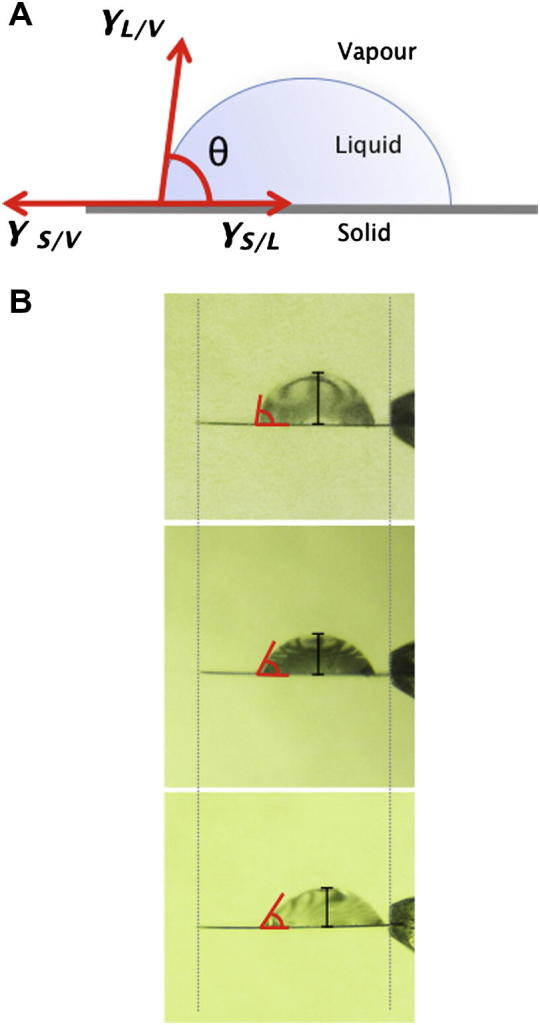
Surface wetting by detergent treatment. (A) Diagram illustrating Young’s relationship for a drop sitting on a surface, which describes the equilibrium established between the solid–liquid (*S/L*), liquid–vapour (*L/V*) and solid–vapour (*S/V*) inter-facial energies (*γ*). (B) Measurements of the contact angle, *θ*, created by a drop of water sitting on non-glow discharged (*top*), LDAO washed (*middle*) and glow discharged grids (*bottom*).

We tried to understand how the detergents might be acting. The ion bombardment of grids by glow discharging aids sample spreading by increasing the strength of interactions between the water molecules in the sample buffer and the surface of the grid, i.e. the wettability of the grid surface. The wettability of a surface can be gauged by the contact angle, *θ*, created between the surface and a drop sitting on the surface, which is defined by the equilibrium established between the inter-facial energies of the three phases: solid (*S*), liquid (*L*) and air (*V* for vapour). In its simplest form, the equilibrium of the inter-facial energies are related by Young’s equation:

$$\gamma_{S/L}+\gamma_{L/V}\cos\theta =\gamma_{S/V}$$

whereby *γ* are the energies at the solid–liquid (*S/L*), liquid–vapour (*L/V*) and solid–vapour (*S/V*) interfaces (Fig. 2). Thus, for a drop sitting on the surface in equilibrium, an inverse relationship is established between the contact angle *θ* and *γ_S/L_*, with a lower *θ* indicative of stronger interactions between the surface and the drop. For a drop of water sitting on an LDAO treated grid, the contact angle was found to be approximately equivalent to that of a drop sitting on a glow discharged grid (50° vs 48°), which was much smaller than that of a drop sitting on a non-glow discharged grid (87°; Fig. 2). This suggests that one main way in which the detergents work is by aiding sample spreading by increasing the interactions between the grid surface and water molecules in the sample.

## 14 Conclusions

The NC, FliF ring and HBB are transmembrane macromolecular complexes with different biochemical properties and purified using different protocols. Yet, when applied to a glow-discharged holey carbon grid, they all demonstrate the same behaviour: adsorption onto the carbon support. It is therefore probable that a multitude of other complexes behave similarly. Indeed, grid manufacturers recognise this problem and offer holey/thin carbon film grids. These comprise of a holey carbon grid onto which a very thin film of continuous carbon is layered. The sample is applied to the surface of this continuous carbon film and vitrified. Sample will spread uniformly over the continuous carbon and, because the carbon film is thin, minimal noise will be added to the signal. However, particles still bind to the carbon in a limited number of orientations, reducing the number of views attainable. Furthermore, the continuous carbon still adds noise to and reduces the signal of the particle images. Particles dispersed in the ice within the holes remains crucial to achieve high-resolution structural analysis.

Grid hydrophilisation by means of detergent treatment is demonstrated here to be an effective method of increasing particle dispersion in the vitreous ice film for cryoEM. In our study, CTAB and/or LDAO had the greatest effect on particle dispersion with most transmembrane protein complexes tested showing increased dispersion, coupled with a reduction of protein adsorbed to the carbon surface. DDM also showed a significant increase in particle dispersion for the NC and HBB samples, although protein still adsorbed to the carbon surface.

Why might LDAO and DDM pretreatments have different effects, given the macroscopic neutrality they both should provide (Table 1)? DDM has a large head group with multiple hydroxyl functional groups, whilst the LDAO head group is comparatively small. It is possible that the hydroxyl groups are forming hydrogen bonds with the protein, which could explain the adsorption seen. If this is the case, then LDAO may be especially useful because it does not have a high potential to form hydrogen bonds. Evidently however, a macroscopically neutral surface is not essential to the success of the procedure, since the cationic detergent CTAB produced excellent results when used with HBBs. These differences may be due the specific overall charge of each solubilised transmembrane protein complex. As this charge cannot be predicted or measured, we recommended that a collection of detergents with different properties, such as the group assembled for this study, is tested for each sample investigated.

On CTAB pretreated grids, deposited NCs show a significant degree of aggregation, but this was not the case for HBBs. Aggregation is reduced with grids pretreated with LDAO for NCs and HBBs, but less substantially for HBBs when applied to DDM-treated grids. Why should this be? When a charged surface is placed into an aqueous environment, an electrical double layer (EDL) forms at the interface between the surface and the liquid. The EDL results from primary ions adsorbed directly onto the charged surface (layer 1) and a diffuse layer composed of solvated ions weakly attracted to the surface (layer 2). The EDL produces a zeta surface potential extending into the liquid orthogonal to the surface. A protein entering this potential is subject to electrostatic forces that can destabilise the various bonds and forces involved in protein folding, leading to degradation and aggregation. The effect of zeta surface potentials on macromolecular complexes might be particularly profound given the array of forces holding them together. A neutral carbon support would not produce an EDL, explaining the reduced protein aggregation seen with LDAO and DDM (Dubochet et al., 1985). However, for particularly stable samples, such as HBBs, this may not be of relevance and in this case, the surface charge generated by the CTAB may actually be beneficial in that it repulses the sample best. This again demonstrates the need to try several different types of detergents for each new sample.

The precise mechanism by which detergent pretreatment increases particle dispersion is not clear. Evidently, enough detergent must bind the carbon surface to lower the surface tension of a buffer drop. Therefore, although the carbon surface of the grid is washed in water after overnight incubation with the detergent, some bound detergent must therefore also leach into the sample buffer. This will increase the overall detergent concentration in the sample and may play a role in particle dispersion but also in protein aggregation. Indeed CTAB, as an ionic detergent and LDAO as a zwitterionic one, are considered harsh and intermediately mild detergents, respectively (Anatrace). However, the non-ionic detergent DDM is considered very mild and is often found to best preserve the biological activity of transmembrane protein complexes. This means that, when considering which detergent to choose for grid pretreatment, a compromise between its effect on grid absorption and self-aggregation/functionality of the sample must be considered. Whatever the mechanism and despite the need to optimise detergent choice for each sample tested, the method presented here provides an alternative to long-standing techniques for cryoEM grid preparation that may prove beneficial for others struggling to obtain dispersed particles.

## Figures and Tables

**Table 1:** Summary of the properties of the detergents used in this study and of their proposed adsorption to a hydrophobic surface.
